# A Case-Control Study of the Fokl Polymorphism of the Vitamin D Receptor Gene in Bulgarians With Lumbar Disc Herniation

**DOI:** 10.7759/cureus.45628

**Published:** 2023-09-20

**Authors:** Lyubomir Gaydarski, Ivo Sirakov, Konstantin Uzunov, Mihail Chervenkov, Teodora Ivanova, Raina Gergova, Ivan Angushev, Georgi Mirazchiyski, Boycho Landzhov

**Affiliations:** 1 Department of Anatomy, Histology, and Embryology, Medical University of Sofia, Sofia, BGR; 2 Department of Medical Microbiology, Medical University of Sofia, Sofia, BGR; 3 Department of Neurosurgery, Pirogov University Hospital, Sofia, BGR; 4 Department of Cytology and Histology, University of Forestry, Sofia, BGR; 5 Department of Biodiversity and Ecosystem Research, Bulgarian Academy of Sciences, Sofia, BGR

**Keywords:** bulgarian population, foki polymorphism, vitamin d receptor gene polymorphisms, low back pain, lumbar disc herniation

## Abstract

Background: The present study investigates whether vitamin D receptor (VDR) gene polymorphisms play a role in intervertebral disc degeneration (IDD), a common cause of low back pain (LBP) and reduced quality of life. Specifically, we examined the FokI VDR polymorphism and its potential association with lumbar disc herniation (LDH) in patients from Bulgaria. Previous studies have suggested a link between mutations in the VDR gene and IDD.

Methods: We investigated whether a potential connection between VDR polymorphisms and LDH was present by comparing the FokI polymorphism of 60 selected patients (25 to 60) with LDH and 60 healthy volunteers within the same age range. We used polymerase chain reaction to assess the phenotype of the examined subjects and statistical tests to evaluate whether the obtained results were statistically significant.

Results: The performed genetic and statistical analyses reviewed significant differences in genotypic frequencies between the patient group and healthy volunteers. The frequency of the F allele is notably higher in patients with LDH (80%) compared to volunteers (52%), while the f allele is more common among patients (86.6%) than volunteers (100%).

Conclusion: This study strongly suggests that expression of the F allele of the VDR gene may increase the susceptibility of developing LDH, while having the f allele could potentially have a protective effect. Our results shed light on the underlying complex mechanisms contributing to the development of LDH.

## Introduction

Low back pain (LBP) commonly causes work disability [1]. The intervertebral discs comprise the outer-annulus fibrosus and inner-nucleus pulposus [2]. The annulus fibrosus comprises several laminae of fibrocartilage, mainly containing type I and type II collagen. Predominantly, type I collagen is found around the ring's edges, as it increases strength in that region. On the other hand, the nucleus pulposus consists of loose collagen fibers suspended within a gel-like substance composed of proteoglycans. The primary function of the nucleus pulposus is to serve as a shock absorber [3]. The prevalence of intervertebral disc degeneration (IDD) in modern society has led to an increase in cases of LBP, with IDD being recognized as the primary contributing factor [1]. The natural aging and degradation of the intervertebral disc results in physiological and pathological changes, leading to various spinal degenerative disorders, including lumbar disc herniation (LDH). Symptoms of these disorders include neck, shoulder, waist, and leg pain, which are common in 80% of adult cases [4]. Nevertheless, the exact etiology of IDD and LDH is yet unclear and is often regarded as multifactorial. The main factors include age, weight, sex, and heavy lifting [5]. However, recent studies imply that LDH has a genetic predisposition [6-9].

Vitamin D is essential for maintaining the body's calcium and phosphorus homeostasis. Recent research has indicated that low vitamin D levels can lead to LBP [10], whereas vitamin D supplementation can alleviate musculoskeletal pain and enhance strength [11]. It has also been noted that vitamin D can influence the metabolism of the annulus fibrosus and nucleus pulposus and the conversion of collagen types I and II, which consequently points to its potential role in LDH [12]. The vitamin D receptor (VDR) gene, situated on chromosome 12q12-q14 and consisting of eight protein-coding and six untranslated exons, has been extensively researched as a potential candidate gene linked to disc degeneration [13]. Yang et al. demonstrated, via immunohistochemical staining, the presence of VDR in the intervertebral disc [14]. Variants of this gene, such as TaqI (rs731236), FokI (rs2228570), and ApaI (rs7975232), have been investigated for their association with IDD [6,10,14]. For the FokI gene, there are two known versions: the F allele codes a 424 amino acids (AA) protein, whereas the f allele codes a 427 AA protein [15]. There are contradicting reports throughout the literature, as several studies suggest genetic predisposition [6-8,16], and others refute it [12,17,18]. Concrete evidence of correlation between VDR gene polymorphisms and IDD, including LDH, remains elusive, regardless of all prior studies. Therefore, further research on this complex conundrum is necessary to clarify the role of vitamin D and VDR in the pathogenesis and progression of IDD. Thus, the present study aims to investigate a potential association between LDH and FokI polymorphism in the VDR gene in the Bulgarian population. The economic and social burden brought about by LBP and LDH is significant. Therefore, it is crucial to comprehend better the sophisticated etiology of this complex pathology, which would pave the way for developing new treatment methods and preventing LDH and LBP.

## Materials and methods

Study design, samples, and inclusion and exclusion criteria

The present work is designed as a case-control study, including 120 people: 60 patients with clinically manifesting and MRI-proven LDH and 60 healthy volunteers serving as a control group. Inclusion criteria include patients with MRI-proven LDH and clinically manifesting symptoms. We examined 60 blood samples from Bulgarian patients aged between 25 and 60, diagnosed with disc herniation, all surgically treated in the UMHATEM "N. I. Pirogov" between 2020 and 2022. For controls, we collected 60 blood samples from clinically healthy people from the same age group with no family history of lumbar disease. The blood samples were obtained sterilely from the median cubital vein. The samples were collected and stored in vacuum containers with EDTA or we used GENO CARD (Hain Lifescience, Germany). We excluded old patients (over 60 years of age), obese patients (BMI over 30), and patients who declared smoking.

DNA, polymerase chain reaction, and restriction enzyme analysis

We obtained DNA using the ISOLATE II Genomic DNA Kit (Bioline, UK), and for conducting polymerase chain reaction (PCR), we used MyTaq™ Mix (Bioline, UK). We performed PCR and restriction enzyme analysis as described by Vieira et al. [7]. To establish the FokI/T2C/rs222857 polymorphism in the VDR gene, we performed PCR with a primer concentration of 10 pmol/uL forward: 5'-AGCTGGCCCTGGCACTGACTCTGCTC -3' and reverse: 5'-ATGGAAACACCTTGCTTCTTCTCCCTC-3' (Jena Bioscience, Germany), 500 ng/uL DNA template, 25 uL BIO-X-ACT™ Short Mix (Bioline, UK) and up to 50 uL PCR water (Bioline, UK). We used the following thermal profile: heated lid 112 C, initial denaturation 95 C - 3 minutes, 35 cycles: denaturation 95 C - 0.45 minutes, annealing 68.0 C - 0.45 minutes; elongation 72 C - 1 minute, final elongation 72 C - 7 minutes, store at 10 C, using FluoroCycler® 12 (Hain Lifescience, Germany). We purified the obtained PCR products using a PCR purification kit (Jena Bioscience, Germany). We analyzed the restriction enzyme with the FokI enzyme (New England BioLabs, USA). We controlled the quantity and quality of the obtained DNA by GeneQuant II DNA/RNA calculator (Pharmacia Biotech, UK) needle electrophoresis with 2% agarose (Bioline, UK), ethidium bromide 1 μg/ml (Sigma-Aldrich, EU), 1×TBE buffer, 120 V, 50 mA for 40 minutes. A 100 bp molecular weight marker (Bioline, UK) was used.

Statistical analysis

Mann-Whitney U test was used to assess the frequencies of healthy controls and symptomatic patients between sexes, whereas the Kruskal-Wallis test was applied for comparisons across ages. Allele and genotype frequency differences between control and LDH-affected groups were analyzed using Fisher's exact test. Only differences at p-values <0.05 (double-sided) were considered significant.

## Results

**Figure 1:**
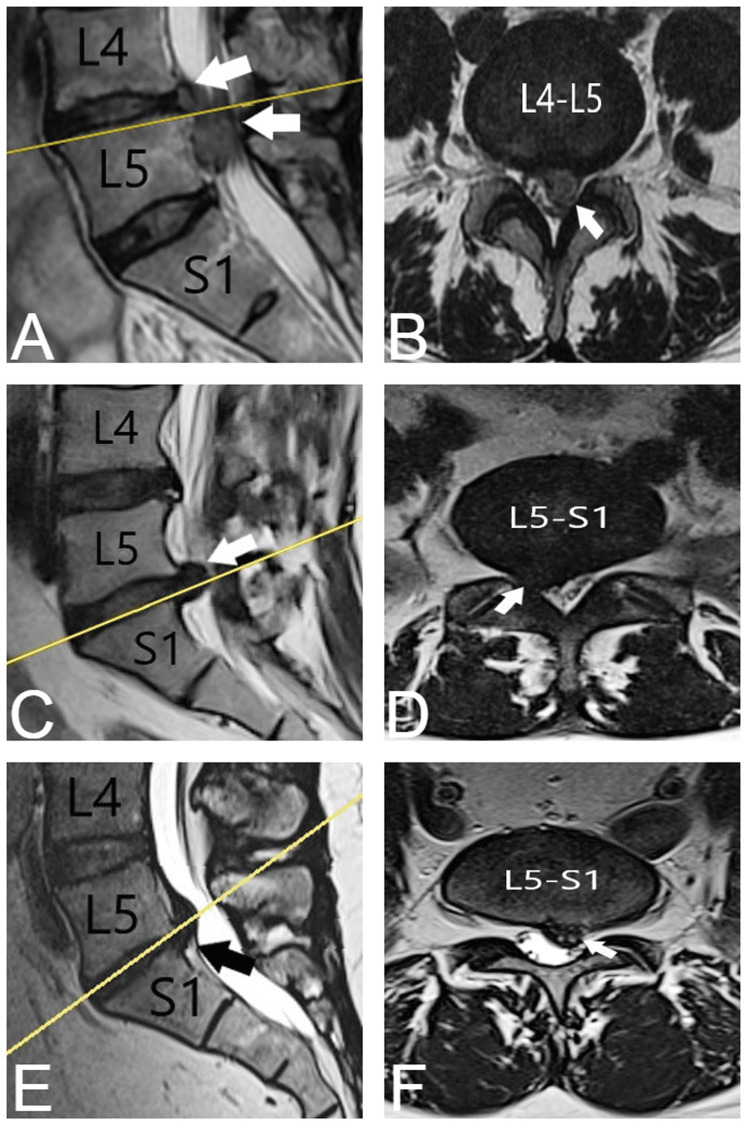
MRI results of patients with LDH (A) Sagittal T2-weighted MRI displaying disc hernia at the level of L4-L5 spinal segment. (B ) Transversal T2-weighted MRI showing a L4-L5 herniated disc. (C) Sagittal T2-weighted MRI displaying L5-S1 disc herniation. (D) Transversal T2-weighted MRI showing a L5-S1 herniated disc. (E) Sagittal T2-weighted MRI displaying L5-S1 disc herniation. (F) Transversal T2-weighted MRI showing a herniated disc at the L5-S1 spinal segment. Arrows displaying the disc herniation.

All patients with symptomatic LDH were diagnosed with the help of an MRI examination. The results of the MRI of three patients with LDH who subsequently underwent surgery are displayed in Figure 1.

Participants' ages varied from 25 to 60 years, with a mean age of 47.55. Moreover, half of the patients diagnosed with LDH were under 50. For clarity, we divided patients and controls into three age groups: under 30, 30-50, and over 50. The number of male patients (n=70, 58.7%) was slightly higher than the females (n=50, 41.3%). However, age and sex were found non-significant for the incidence of LDH, p=0.069 and p=0.712, respectively (Table 1).

**Table 1:** Descriptive statistics for the parameters age and sex of both examined patients and controls N: number of participants, NS: non-significant, LDH: lumbar disc herniation (p> 0.05, age: Kruskal–Wallis test, sex: Mann–Whitney U test)

		Female (NS)	Male (NS)	Total (NS)
N (%)		50 (41.3)	70 (58.7)	120
Age (NS)	Mean ± SD	44.67 ± 4.7	50.43 ± 1.4	47.55 ± 9.3
	Median	43.00	54.00	48.50
Health status	Age group			
Healthy control	Under 30, N(%)	3 (6.0)	0 (0.0)	3 (2.5)
	30-50, N (%)	11 (22.0)	19 (27.1)	30 (25.0)
	Over 50, N (%)	12 (24.0)	15 (21.4)	27 (22.5)
LDH	Under 30, N(%)	0 (0.0)	0 (0.0)	0 (0.0)
	30-50, N (%)	10 (20)	20 (28.6)	30 (25.0)
	Over 50, N (%)	14 (28.0)	16 (22.9)	30 (25.0)

**Figure 2:**
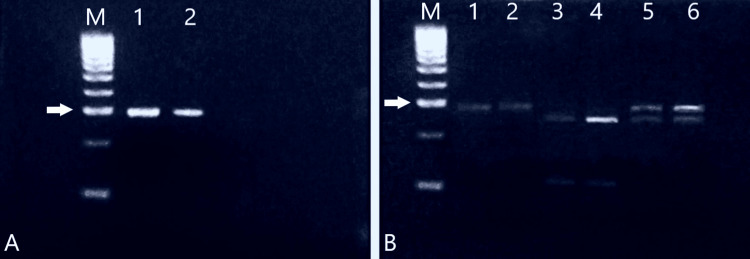
PCR results (A) M–DNA marker across 100 nucleotides (nt), 1 and 2 are samples after PCR and gel purification. (B) Result after restriction analysis: M–DNA marker through 100 nt, 1 and 2 - fragments not cut by the enzyme, homozygous TT, 3 and 4 - homozygous CC, 5 and 6 - heterozygous CT genotype. White arrow – 300 nt

Afterward, we performed PCR of both groups and obtained products weighing about 300 base pairs (bp). The frequency of the F allele of the VDR gene in patients with LDH was 80% (n=48). This value was statistically significantly higher (p=0.002) than healthy volunteers, whose incidence was 52% (n=31). The frequency of the f allele in the healthy volunteers was 100% (n=60), and in the patients with LDH was 86.6% (n=52) (p=0.006). Consequently, the genotypic frequencies of healthy volunteers and patients differed significantly (p<0.0001), with 48.3% (n=29) to 20% (n=12) for TT genotype; 51.6% (n=31) to 66.6% (n=40) for CT genotype; and 0% (n=0) to 13.3% (n=8) for genotype CC for both groups, respectively. The results of restriction enzyme analysis are shown in Figure 2 and Table 2.

**Table 2:** Descriptive statistics for the parameter genotype of both examined patients and controls (%)

	TT	TC	CC	Total
Controls	29 (48.3)	31 (51.6)	0 (0)	60 (100)
Patients	12 (20)	40 (66.6)	8 (13.3)	60 (100)

Furthermore, we calculated the odds ratio (OR) between the different FokI polymorphisms and the likelihood of developing LDH. The OR between the F allele and developing LDH is 3.7419 (95 % CI) with p=0.0014. Thus, it is highly likely that individuals carrying the F allele of the VDR gene are approximately 3.74 times more likely to develop LDH compared to those without this allele. The low p-value (0.0014) suggests statistical significance, meaning this association is unlikely to be due to random chance. Conversely, the OR between the f allele and LDH is 0.0510 (95 % CI) with p= 0.0426, which indicates that individuals with the f allele of the VDR gene have a significantly lower likelihood of developing LDH. In addition, we evaluated the OR between the different genotypes and LDH. The OR between the TT genotype and LDH is 0.2672 (95 % CI) with p=0.0014, which implies that people with this genotype have a statistically significantly lower risk of developing LDH. The OR between the TC genotype and LDH is 0.5345 (95 % CI) with p=0.0961, which suggests that individuals with the TC genotype have a lower likelihood of LDH. However, this result is not statistically significant. Moreover, the OR of the CC genotype is 19.5905 (95 % CI) with p=0.0426, which strongly implies that individuals with the CC genotype have a notably higher risk of developing LDH. This correlation is statistically significant (p<0.05). All results of the OR calculation are presented in Table 3.

**Table 3:** Descriptive statistics of the parameter OR between FokI polymorphisms and the likelihood of developing LDH CI: confidence interval, P: statistical significance (p<0.05)

	OR	CI	z statistic	P
F allele	3.7419	95% ( 1.6642- 8.4139)	3.192	0.0014
f allele	0.0510	95% (0.0029-0.9058)	2.027	0.0426
TT genotype	0.2672	95% (0.1189-0.6009)	3.192	0.0014
TC genotype	0.5345	95% (0.2555-1.1179)	1.664	0.0961
CC genotype	19.5905	95% (1.1040-347.6240)	2.027	0.0426

## Discussion

IDD and LDH have complex and not entirely understood etiology, as currently, the main etiological factors are environmental factors and genetic predisposition [6]. Vitamin D metabolism is essential for calcium and phosphorus homeostasis; thus, it is pivotal for the normal function of the musculoskeletal system [19]. Vitamin D exerts its function via its receptor, the VDR, which has been extensively examined as a potential candidate gene in various musculoskeletal and non-skeletal disorders. Its impact has been demonstrated in conditions such as osteoporosis and elevated risk of fractures [20]. The evidence shows that vitamin D regulates cell proliferation and cytokine production in the lumbar annulus [21]. In addition, genetic polymorphisms of the VDR are linked with alternations of vitamin D metabolism [22]. In a recent study, Colombini et al. reviewed that the FokI f allele of VDR is associated with better coping with inflammation than the F allele [23]. This statement might explain why the F allele is linked with a higher probability of IDD. As several different polymorphisms of the VDR gene are known, the FokI is of particular interest due to its alleged association with the pathogenesis of IDD [6,7,24]. We are the first to investigate VDR polymorphisms' role and LDH's development in the Bulgarian population. Our study suggests a statistically significant correlation between the expression of the F allele of the FokI gene of the VDR and the progression of IDD to LDH. Our findings support several previous studies. Vieira et al. found a dependence between FokI polymorphism, T to C in the VDR, and IDD in an investigation of 121 Brazilian men [7].

Furthermore, a reason study of Yang et al. revealed a statistically significant correlation between TaqI polymorphism and LDH [14]. Zhao et al. performed a meta-analysis demonstrating the increased risk for IDD among those with VDR rs2228570 polymorphism [8]. Zawilla et al. conducted a case-control study with 84 lumbar disc degeneration and 60 control subjects, demonstrating a correlation between VDR Apal polymorphism and the risk of LDH [16]. Fang et al. reported an increased risk of IDD among people with TC and CC genotypes compared to the TT genotype of FokI [24]. Moreover, Biczo et al. reported 5'-(Cdx2, A1012G) mutations of VDR linked with endplate defects and 3'-(BsmI, ApaI, TaqI) with Modic changes, both resulting in a higher risk for IDD and LDH [25]. Xue et al. performed a meta-analysis of 23 studies. They concluded that FokI polymorphisms are linked with the elevated risk of IDD, including LDH, among Caucasians but not among the Asian population [9]. Another meta-analysis of Castillo-Avila et al., including 1,549 patients and 1,672 controls, indicated a statistically significant correlation between FokI polymorphisms and IDD [26]. Lan et al. suggested that VDR mutations might hinder vitamin D metabolism, leading to the modulation of autophagy and the progression of IDD. This might be one plausible explanation for the association between VDR polymorphisms and IDD [27]. On the other hand, several articles deny any statistically significant correlation between VDR genetic variants and the progression or onset of intervertebral disc degeneration or osteoarthritis [12,17,18,28]. Chen et al. and Pabalan et al. reported that FokI polymorphisms reduce the risk of IDD in Caucasians [18,28]. Pekala et al. used the Jadad decision algorithm incorporating the result of seven prior meta-analyses involving 2,580 patients and determined no significant correlation between FokI polymorphisms and IDD [29]. Gao et al. performed a meta-analysis involving 21 studies (2,781 patients and 3,512 controls) and determined no statistical significance between FokI polymorphisms and IDD [30]. The observed discrepancy between the above-stated results might be due to variations in sample size, ethnicity, and differences in the design of the studies.

In addition, the association of VDR polymorphisms and IDD offers significant clinical implications. Yang et al. suggested that plasma levels of VDR might be a reliable marker for the development and deterioration of IDD. Furthermore, knowledge of VDR polymorphisms might serve as a genuine genetic screening method for identifying susceptible individuals before the clinical manifestation of symptoms [14]. The current research paves the way for future investigation of personalized treatment approaches based on an individual's VDR gene polymorphisms, vitamin D plasma levels, and novel therapeutic possibilities for IDD treatment.

Nonetheless, our study has several limitations to consider. Firstly, we examined a small cohort of patients (n=60), which diminished our ability to draw vast conclusions. Secondly, our study only focused on FokI polymorphisms, thus omitting to assess mutations in the other VDR alleles. Lastly, we only evaluated FokI alleles but did not examine VDR and vitamin D plasma levels, which would have provided additional information about the sophisticated mechanisms between vitamin D metabolism and LDH. Nevertheless, our study provides critical insight into FokI polymorphism in the Bulgarian population and their association with LDH and paves the way for future research.

## Conclusions

The study conducted is the first of its kind for Bulgaria, and the data obtained are promising reasons to suggest that the VDR gene polymorphism is linked with the development of LDH in Bulgarian patients. Our study found that the F allele (C) in the VDR gene correlates with greater frequency for developing LDH, whereas the f allele (T) plays a protective role. Our results imply that VDR polymorphisms might serve as a predictive factor for the development and deterioration of LDH. Therefore, such knowledge is essential for the prevention of the illness.
